# Qualitative Interview Studies of Working Mechanisms in Electronic Health: Tools to Enhance Study Quality

**DOI:** 10.2196/10354

**Published:** 2019-05-06

**Authors:** Marianne TS Holter, Ayna B Johansen, Ottar Ness, Svend Brinkmann, Mette T Høybye, Håvar Brendryen

**Affiliations:** 1 Norwegian Centre for Addiction Research (SERAF) Institute of Clinical Medicine University of Oslo Oslo Norway; 2 Department of Education and Lifelong Learning Norwegian University of Science and Technology (NTNU) Trondheim Norway; 3 Department of Communication and Psychology Aalborg University Aalborg Denmark; 4 Elective Surgery Centre Regional Hospital Silkeborg Silkeborg Denmark; 5 Department of Clinical Medicine Interacting Minds Centre Aarhus University Aarhus Denmark

**Keywords:** telemedicine, eHealth, mobile health, telehealth, mHealth, interviews as topic, health care evaluation mechanisms, data collection

## Abstract

Future development of electronic health (eHealth) programs (automated Web-based health interventions) will be furthered if program design can be based on the knowledge of eHealth’s working mechanisms. A promising and pragmatic method for exploring potential working mechanisms is qualitative interview studies, in which eHealth working mechanisms can be explored through the perspective of the program user. Qualitative interview studies are promising as they are suited for exploring what is yet unknown, building new knowledge, and constructing theory. They are also pragmatic, as the development of eHealth programs often entails user interviews for applied purposes (eg, getting feedback for program improvement or identifying barriers for implementation). By capitalizing on these existing (applied) user interviews to also pursue (basic) research questions of how such programs work, the knowledge base of eHealth’s working mechanisms can grow quickly. To be useful, such interview studies need to be of sufficient quality, which entails that the interviews should generate enough data of sufficient quality relevant to the research question (ie, *rich data*). However, getting rich interview data on eHealth working mechanisms can be surprisingly challenging, as several of the authors have experienced. Moreover, when encountering difficulties as we did, there are few places to turn to, there are currently no guidelines for conducting such interview studies in a way that ensure their quality. In this paper, we build on our experience as well as the qualitative literature to address this need, by describing 5 challenges that may arise in such interviews and presenting methodological tools to counteract each challenge. We hope the ideas we offer will spark methodological reflections and provide some options for researchers interested in using qualitative interview studies to explore eHealth’s working mechanisms.

## The Need to Identify the Working Mechanisms of Automated Electronic Health Programs

Building the next generation of automated electronic health (eHealth) programs will require a shift of attention from the performance of individual programs to a joint effort of understanding eHealth’s working mechanisms [1,2]. The term *eHealth* is a broad term that may refer to many forms of technological health support; this paper concerns itself with automated Web-based interventions for mental and physical health purposes [3], or *eHealth programs*. The outcomes of individual eHealth programs are well established; a vast majority of the research published between 1996 and 2013 concerned outcome (74%). However, much fewer publications focused on processes (26%) [4]. Consequently, the working mechanisms that underlie the outcomes of individual interventions are substantially less documented [5-7]. This is not only theoretically unsatisfactory; it is also problematic when it comes to designing new eHealth programs, as there are few, if any, field-specific theories of models that can be used to inform program development.

Instead, the development of eHealth programs often relies on rather static traditional behavior change theories [2] or models from face-to-face therapy [8-11], under the assumption that the principles are transferrable to automated eHealth therapy. However, the interaction between a program user and an automated eHealth program is in many cases *not* static; many programs include different degrees of interactivity and tailoring [3,5], making traditional behavior change theories potentially unsuitable [1,2]. On the other hand, using models from face-to-face therapy may not be appropriate either, as automated eHealth therapy by definition does not involve human contact. As automated eHealth programs are neither static nor involve human contact, it is possible (or even likely) that the way they achieve their effects is not explained with established theories and models [1,2]. This suggests a need for research that can identify eHealth’s working mechanisms, knowledge on which it is possible to build eHealth-specific theories and models.

## Qualitative Interviews: A Promising and Pragmatic Method for Studying Electronic Health Programs’ Working Mechanisms

eHealth’s working mechanisms can be studied using various methods, but a promising and pragmatic venue of investigation is the qualitative interview, that is, “professional conversations (...) where knowledge is constructed in the inter-action between the interviewer and the interviewee (...) about a theme of mutual interest” [12]. The qualitative interview is a promising method for investigating eHealth working mechanisms as it grants unique access to participants’ experiences and as it is especially suited to explore what is unknown [13]. Providing a means to explore the unknown makes qualitative interviews a potent method for generating new knowledge and theory [14,15], and some interview studies have already demonstrated their potential for uncovering important insights about the processes that may be involved in automated eHealth therapy [16-19].

The qualitative interview is also a pragmatic research method, as many researchers already conduct interviews with program users as part of an applied research goal (developing or implementing an intervention). In the process of conducting interviews with program users, a researcher may become intrigued by a more basic research question and may perhaps consider the pragmatic solution of pursuing both the applied and the basic research goal in the same interviews by simply adding questions to the existing interview guide. We believe that such studies mixing basic and applied research goals have the potential of becoming an important asset to the field, by accumulating knowledge on more general issues that may help us understand how eHealth therapy works.

However, to become such an asset, the interviews conducted in these studies should provide what in qualitative methodology is known as *thick descriptions* or *rich data* [20,21]. Rich data are usually considered a requirement for a valid qualitative analysis, and the concept signifies having enough data of sufficient quality relevant to the research question, including both variation (ie, data breadth) as well as details and nuances (ie, data depth) [22,23]. Data that are *not* rich—that lack in breadth or depth—might threaten the study’s quality or the potential reach of its conclusions. Thus, getting rich data that inform the research question is an important aspect of a qualitative study. In the case of qualitative interviews, producing rich data means conducting interviews in a way that makes the participants spend a lot of time talking about the aspects that are central to the investigation, including both breadth and depth in their descriptions. This may seem straightforward, but it can be surprisingly difficult in practice.

## Difficult in Practice: The Case of a Study on a Person-To-Program Alliance

The reflections that are presented in this viewpoint paper arose from some of the authors’ experiences with a specific interview study [24] (in review), the aim of which was to explore a potential person-to-program alliance. The study in question included the development of an alliance-supporting program [25], and in an early study phase, the interviews had both an applied and a basic purpose: the applied purpose was getting feedback for program improvement, and the basic purpose was exploring how the participants related to the program. By exploring how participants related to the program, we hoped to achieve a better understanding of a person-to-program alliance [26-28] as a potential eHealth working mechanism. However, it was surprisingly difficult to conduct interviews that would yield rich data on how the participants related to the program, and the initial interviews resulted in scant data to answer the research question. This left us with 3 options: (1) answering the basic research question with scant data, which would limit the conclusions we could draw, (2) abandoning the basic research question as unanswerable, or (3) trying to generate richer data by changing the way the interviews were being conducted.

We opted for trying to improve the interview method; however, we found no guidelines within the field of eHealth for how to conduct high-quality qualitative interview studies on potential eHealth working mechanisms. Therefore, we started looking more closely at the interviews we had conducted, asking ourselves what had gone wrong. This process led to the identification of a handful of problems that we believed were likely to have contributed to the difficulties in getting rich data on how the participants related to the program. As we started defining these problems, we discovered that we had also encountered several of them in other eHealth studies we had been involved in [29-33], and we therefore believed they could be relevant beyond the specific study we were currently engaged in.

We wanted our experiences to be of benefit to other researchers with similar agendas and interests, and we therefore sought to describe the problems we had encountered in a way that would maximize their generalizability. Thus, through discussion among ourselves and with other researchers, we conceptualized 5 interview challenges: achieving a joint understanding of the interview topic, keeping participants from straying off the focus of enquiry, aiding recall of specific program experiences, avoiding negative influence of the social interview situation, and structuring the dual-aim interview. Having identified the challenges, we consulted the literature on qualitative methodology to identify methodological tools to counteract each challenge.

Returning to the study that had started this process [24], we changed the interview method to include some of the tools we had identified. This markedly enhanced the quality of subsequent interviews, producing rich data to answer how the participants related to the eHealth program as well as whether this way of relating influenced change. Thus, although the original interview method generated scant data on the basic research question, the revised interview methodology led to interviews that could answer the same research question with rich data.

In short, although it seemed a pragmatic solution to use already-planned interviews to pursue the answer to a basic eHealth research question, we experienced that getting rich data on the basic research question was challenging. In the absence of guidelines for conducting high-quality qualitative interviews specifically adapted to the field of eHealth, the process we entered into led to an enhanced methodological awareness and specific methodological tools for increasing study quality. The main focus of this paper is to share the identified challenges and tools with the research community. However, before doing so, we will offer what we consider to be a handy heuristic for understanding some of these methodological challenges: the *invisible interaction* between eHealth program and program user.

## A Handy Heuristic: The Invisible Interaction

**Figure 1:**
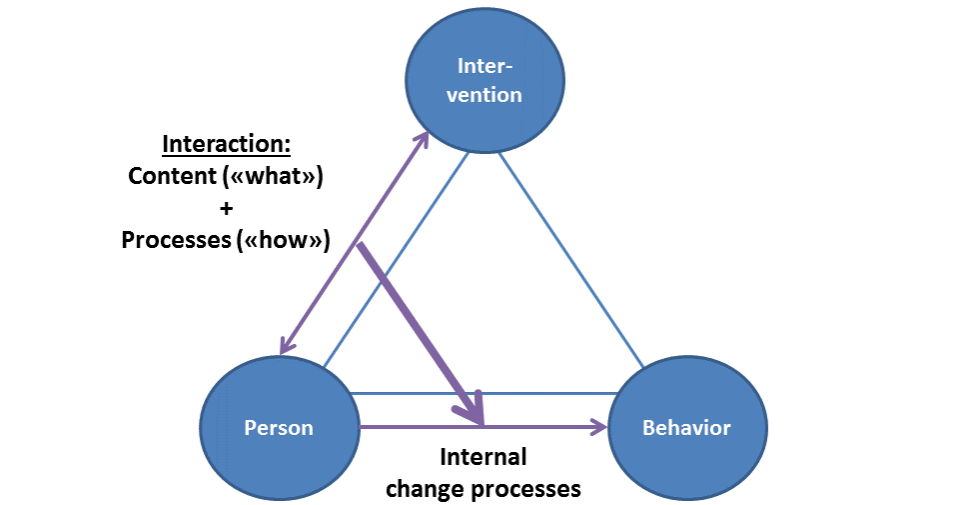
Working mechanisms of a behavior change intervention.

We suggest that a person’s interaction with any health intervention can be visualized in terms of a triangle, which includes the individual help seeker, the intervention, and the behavior change processes (Figure 1, adapted from Moen and Middelthon’s discussion of interviews) [34]. A health intervention’s working mechanisms can be conceptualized as how the interaction between the person and the health intervention influences the person’s internal change processes. The interaction, in turn, can be described as a combination of the interaction’s content (the *what* of the interaction) and the interactional processes (the *how* of the interaction). For example, psychotherapy’s working mechanisms can be described as the therapy sessions’ influence on the client’s internal change processes. The therapy sessions, in turn, can be described as comprising 2 main elements: their content (eg, the topic discussed) and the interactional processes, when and how often interaction is initiated, how the interaction unfolds, how the next interaction is initiated, and so on.

However, interventions may differ according to how much the 2 interacting parties—the person and the intervention—influence the interactional content and the interactional processes. In the case of psychotherapy, both the client and the therapist highly influence both components of the interaction. Taking another example, a person reading a self-help book is also interacting with a health intervention: things also *act*, and people interact with them, in that the properties of a thing influence how a course of action involving that thing unfolds [35]. The working mechanisms of a self-help book can therefore also be described in terms of the interactional content and the interactional processes. However, the relative influence of the 2 interacting agents (reader and book) differ from the case of psychotherapy. The book decides the interactional content, although the reader largely decides the interactional processes: when and how often interaction is initiated (when to read again), how the interaction unfolds (what to read in what sequence), how the next interaction is initiated (picking up the book), and so on.

**Figure 2:**
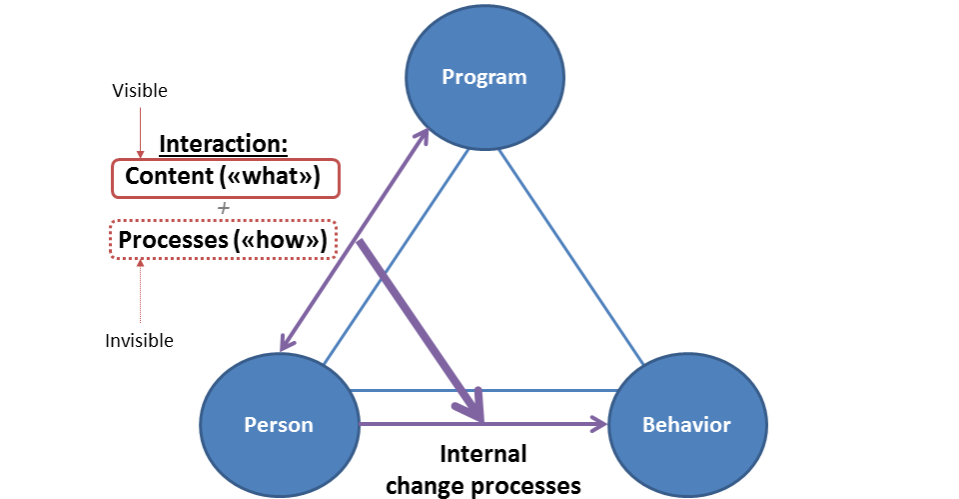
Working mechanisms of an automated electronic health intervention.

Considering the working mechanisms of an eHealth program, many programs will influence both the interactional content and the interactional processes. As with a self-help book, the interactional content will usually to a large extent be decided by the program. Moreover, just as a self-help book, the program is a *thing*, and many people are likely to think of things such as computer programs as inanimate objects with content. Indeed, the most prominent feature of eHealth programs is their content [3,10], even though they also may substantially influence the interactions with the user [3]. For example, eHealth programs may influence when and how often the interaction takes place (eg, through reminders to log on), how the interaction unfolds (eg, by responding with tailoring to user input), how the next interaction is initiated (eg, through invitation), and so on [5,10]. Some of these interactional processes may not be experienced directly by the individual user, for example, in the case of tailoring, the program may be adapted specifically to the user’s input, but she or he nevertheless only sees 1 version of the program, masking the actual interaction. In sum, although eHealth programs may have a substantial influence on the interactional processes, the average program users may primarily focus on their content and think of them as inanimate objects that do not interact. In other words, to the user, the interaction with the program can be largely invisible (Figure 2).

The invisible interaction is a useful heuristic when considering the challenges of interview studies for exploring eHealth’s working mechanisms. We previously stated that an asset of qualitative interviews is their potential to explore eHealth working mechanisms from the program user’s perspective. However, from this perspective, part of the program’s working mechanisms—the interactional processes— are maybe invisible to the participant, unless she or he purposefully directs his or her attention toward them. In other words, being largely invisible, the interactional processes may not be part of the participant’s conscious experience that she or he is ready to share in an interview. This may create or contribute to certain challenges with exploring eHealth working mechanisms through interviews. We will now present 5 such challenges and suggest methodological tools to counteract them.

## Interview Challenges and Tools

### Achieving a Joint Understanding

When a researcher sets out to explore a potential eHealth working mechanism in an interview, it may be difficult to achieve a joint understanding of the interview topic together with the participant. For example, as mentioned previously, in the study that was the starting point for this paper, the researchers were interested in understanding how the participants related to the program [24]. The interview guide comprised mainly of descriptive interview questions—questions that ask the participant to describe a specific experience, which are usually recommended to get close to the participants’ own experience [13]. Examples of descriptive interview questions were *could you tell me what you thought and felt the first time you used the program* and *could you describe the role the program has had in your quit attempt* (the program was for helping people quit smoking). However, the researchers struggled with superficial answers that did not seem to reveal anything about how the participants related to the program (such as *I thought the program was fine*) until 1 participant called the program *a secret friend*. The researchers were puzzled. Was this person’s program experience unique? Why did other participants not talk about the program in this way at all? However, apart from a few statements similar to this one, the interviews were not generating data to answer the research question of how the participants related to the program.

Failure to get rich data on a research question may indicate a marginal phenomenon—or that the interviewer is failing to communicate the focus of enquiry in a way that facilitates joint understanding with the participant. We believe it is a truism that experience is multifaceted and that an experience can be described from many perspectives. For example, a client may describe a therapy session from a factual perspective of when and where it took place, from an experiential perspective of his or her emotions before, during, and after the session, from a historical perspective of the session as a stage in his or her spiritual development, and so on. If the interviewer’s questions are mostly descriptive, there may be a scarcity of cues concerning which perspective to assume, leaving the decision up to the participant—and the participant’s choice may not be the researcher’s choice. This may be especially challenging in studies on eHealth working mechanisms, as the interactional processes may not be part of the participant’s conscious experience. Therefore, descriptive questions asking for the participant’s program experiences will perhaps not cause him or her to talk about the (invisible) person-program interaction but rather about the program as a thing with a content. On the other side of the conversation, the interviewer may fear that more direct questions onto the focus of enquiry will put words in the participant’s mouth and disqualify any subsequent answer.

An interviewer can use several methodological tools to foster a joint understanding of the interview topic with the participant. One such tool is vignettes: vivid, exemplifying prose stories that guide the conversation toward a particular aspect of the participant’s experience [36-38]. The interview vignette is constructed before the interviews and included in the interview guide. The vignette can be constructed on the basis of a participant account, on relevant literature, or on the researcher’s current understanding of the processes under study. The interviewer might introduce the vignette by saying that she or he wants to share a story with the participant. After recounting the vignette, the interviewer can ask for the participant’s reactions and ask follow-up questions (eg, if the participant has experienced anything similar or can provide a different perspective). Using several vignettes in the same interview can be a useful way of illustrating different perspectives on the research topic. This will implicitly communicate to the participant that all answers are acceptable, ensuring that the vignettes function to guide the conversation but not restrict the answers [36,38].

Another and more direct way of fostering a joint understanding of the interview topic is to involve the participants as coresearchers, or using epistemic interviewing [13,14,39,40]. In traditional qualitative interviewing, participants describe their subjective experiences and the data are analyzed and interpreted afterward by the researcher [13,14]. In contrast, a coresearcher design entails that the researcher shares his or her current understanding of the research topic and asks for the participant’s views, and the research questions are investigated in collaboration. Involving participants as coresearchers also changes the roles of the interviewer, who becomes a sort of participant contributing with his or her perspective. This joint exploration entails that much of the analysis and validation is done in the interview [14,39].

A final tool to clarify and exhaust the interview topic is to ensure the possibility of conducting follow-up interviews [41]. A follow-up interview gives both the interviewer and the participant an opportunity to reflect on what was talked about in the first interview, allowing new insights or aspects to emerge [22]. It also gives the researcher an opportunity to clarify questions or test interpretations with the participant directly [39], giving more nuanced data and enhancing validity.

Coresearcher design and vignettes foster clearer communication, but they may also threaten the study’s validity if the researcher holds on to his or her initial assumptions about the studied process, failing to acknowledge unexpected perspectives. To ensure that these tools strengthen and not hamper the quality of the study, the researcher should adopt what in psychotherapy is known as the *beginners mind*: remaining curious and receptive, open to all possibilities [42-44]. Furthermore, the interviewer must throughout the research process practice reflexivity, that is, considering how she or he may be affecting the study with “(…) thoughtful, conscious self-awareness” [44]. Reflexivity about, for example, preunderstandings, motivations, and the influence of previous experiences can lead to important insights [43,44]. Reflexive insights that could be of importance for analysis should be documented (eg, through memos or notes) [15,43,44]. Finally, documented reflections should be made part of the analysis and be made explicit to the reader [44].

### Keeping Participants From Straying off the Focus of Enquiry

To allow time for joint exploration of the person-program interaction, it is necessary to limit the interview time spent on matters that are not at the core of the research question. Returning to Figure 2 and the triangle of program, user, and behavior change, the relative importance of each triangle endpoint will vary according to the research question: some parts of the triangle will be the focus of enquiry, whereas the other parts will be contextual. For example, in 1 study [33] (paper under preparation), the researchers interviewed patients who had gambling problems and had used a Web-based referral site to connect with problem gambling services. The focus of enquiry was their use of the website; the gambling problems were the context. However, the interviewer struggled with keeping the conversation focused on the website, as participants talked mostly about their personal history with gambling problems. When asked about their experiences with the website, they appeared to feel alienated and at a loss. Consequently, there was a lot of interview data on the participants’ behavior change efforts—but little data on their use of the website.

If the participants continuously stray off the focus of enquiry by spending time on contextual aspects, it can threaten the data richness. Aspects that are contextual to the researcher may be aspects the participant wants to share or aspects she or he believes to be important to the investigation. The interviewer may try to lead the conversation back onto the focus of enquiry, but the participant may return to the contextual aspects, turning the interview into a battle over topic. Apart from being unpleasant for both, the result may be scant data on the focus of enquiry. When the focus of enquiry is potential eHealth working mechanisms, the invisible interaction may add to the challenge of straying off the topic. As the participant may be largely unaware of the interactional processes, she or he will instead talk about the aspects of which she or he is aware: the change processes (in isolation of the program) or the program (in isolation of the change processes). Information about the behavior change and about the program is certainly relevant contextual information, but talking about these aspects in isolation should not dominate the interview.

The interview conversation can be kept from straying off the research topic by using in-interview questionnaires to keep contextual answers short. The questionnaire can include questions addressing contextual issues (eg, *How long have you been worried about your gambling?* Or *Have you tried restricting how much you gamble before?*), together with any other questions that might serve as relevant analytic background (eg, demographics). The interviewer may fill out the questionnaire together with the participant at a suiting point during the interview. Using a piece of paper to fill out the answers will help keep the answers short, by providing limited space and communicating a wish for answers that the interviewer can write down. Short contextual answers will in turn leave more time for the focus of enquiry.

### Aiding Recall of Specific Program Experiences

Sometimes participants may not recall program experiences in sufficient detail to answer the interviewer’s questions. In the study that inspired this paper [24], the interviewer asked the participants to tell her about a program session they remembered especially well, thinking that she would use this session as a starting point for further descriptive interview questions [13]. To her surprise, several participants who were still active program users and had completed most sessions up until the time of the interview had difficulties remembering any particular program session at all.

Recalling specific program experiences may be challenging as although participants may be active program users at the time of the interview, they are not engaging with the program at that particular moment (unless you are combining the interview with a *think-aloud*-technique, discussed below) [45]. That means that to talk about program experiences, the participants must retrieve memories. However, program sessions may be short, and the participants are likely to use the program in between their other daily business. Consequently, program use may not be encoded as distinct episodic memories to begin with [46]; rather, these memories may be intertwined with other memories of everyday life. Thus, if the interviewer asks the participant to describe a program session, his or her question may not contain the right memory cues [47] to trigger memories of program use, and the participant may seemingly not recall any sessions at all. The invisible interaction may amplify this problem: if the participant is unaware of the program influencing the interaction, these program aspects will be even more difficult to retrieve on demand.

There are, however, methodological tools to amend the problem with recall in the interview situation: 1 such tool is to get *live* access to the person-program interaction through the think-aloud procedure [45]. In the think-aloud procedure, the participants go through (parts of) the program during the interview as the interviewer instructs the participant to *think aloud*, reporting all thoughts without censoring them. The interviewer should not interrupt the participant’s flow of thoughts, and follow-up questions should be saved for after the think-aloud procedure is completed [45]. However, there are some limitations to this approach: unless the program comprises just 1 website or session, the researcher cannot use the think-aloud procedure to go through all program content, requiring him or her to select the most relevant sessions. Furthermore, when the focus of enquiry is working mechanisms within the invisible person-program interaction, the interviewer’s presence may draw attention from the program’s role in the interaction, adding to its invisibility. However, if these issues do not apply, the think-aloud procedure can enable a researcher to study possible eHealth working mechanisms as they happen, potentially removing the problem of recall.

Another tool for aiding recall is asking memory-facilitating interview questions. If program experiences have not been encoded as specific episodic memories, the interviewer’s phrasing of questions becomes increasingly important, as the words she or he uses will influence the participant’s memory-retrieval process by serving as memory cues [47]. The interviewer’s choice of words can be guided by mapping the participant’s program habits early in the interview. Knowledge of program habits can in turn be used to phrase questions in ways that contain memory cues; reflecting what the participant was doing before using the program, where she or he was, and his or her emotional state at the time of the experience [47]. Such memory-facilitating interview questions may help the participant disentangle the recall of program experiences from everyday life.

As a final note on program recall, it may not be necessary for the participant to remember any particular program session at all; the researcher must consider what level of detail is necessary to answer the research questions meaningfully. For some research questions, the sum of program experiences may be more important than any particular experience. If so, using the interview to discuss the participant’s overall experience with the program can be more meaningful than facilitating recall of specific sessions [22].

### Avoiding Negative Influence of the Social Interview Situation

All interviews are also social situations, and aspects of the social situation will influence the data [48]. In 1 of the interviews from the study that inspired this paper [24], a female interviewer interviewed a male participant, with the goal of understanding how he related to the eHealth program he had used. The interview was brief and disappointing; the participant’s answers were short, and the topic was exhausted quickly. It was not until later that the interviewer became aware that she had been afraid of the participants judgment; that he would perceive her as a *typical woman*, valuing emotions (interactional processes; relating to the program) over facts (the program content). This subconscious fear had caused her to rush through the questions (which she during the interview had found awkward), partly answering some of them on behalf of the participant and ending the interview early.

Gender stereotypes are not the only potential social disturbances in an interview—other social roles may be prominent, and within eHealth research, the interviewer may be particularly prone to be perceived as an interviewer or clinician or interviewer or developer. Perceiving the interviewer as also a clinician may cause the participant to think of him or her as a therapeutic interactional partner and to be less attentive to the therapeutic agency of the eHealth program. Similarly, perceiving the interviewer as also a program developer may highlight the program as a thing made by someone else, making it more difficult to see the program’s role as a therapeutic agent—or cause the participant to self-censor negative experiences, as 1 of the authors experienced in 2 different studies [29,31]. In both cases, the interviewer’s presence may cause the participant to think of the *interviewer* as the interacting agent, pushing the experience of the *program* as an interacting agent to the background and adding to the interaction’s invisibility. In sum, the social interview situation may cause the participant to talk differently about his or her program experiences than she or she would have otherwise. The consequence of this may be less rich data, or data that do not correctly represent the participant’s experience.

The potentially negative influence of the social interview situation can be counteracted with methodological tools. The researcher acknowledging the potential negative influence of roles and stereotypes, both before and after the interviews, can minimize their negative effect. Before an interview, researchers should reflect on potentially salient social aspects and whether something should be done about them [43]. If circumstances can make the interviewer appear as a clinician or a program developer, the interviewer may try to change these circumstances beforehand, for example, by changing the interview location or considering how to dress or talk. Alternatively, these issues can be addressed explicitly in the beginning of the interview, clarifying the interviewer’s role [19]. During the interview, the interviewer should try to monitor the social exchange [43], making notes of elements that may be impacting the conversation. After the interview, anything that might be of importance to the analysis should be documented [44]. These notes should be included somewhere easily accessible (eg, in the interview transcript or in a separate document) and analyzed as data that might inform, confirm, or qualify the analysis. Regarding the danger of additionally concealing the invisible interaction through the social exchange between the interviewer and the participant, the interviewer can try to arrange the interview situation so that it includes all 3 as potential agents: the participant, the program, and the interviewer [34].

Finally, it is important to acknowledge that although the social interview situation may sometimes be a negative influence on the data, it can also be an asset. Through the interviewer’s reflexivity, the social situation may generate insights that would otherwise be missed. The interview in which the interviewer had rushed through the questions as she feared being labeled an *emotional woman* was considered as empirical material highlighting a possibly relevant aspect of how people *relate* to a program, namely, that relating to a program may go against social norms and produce feelings of embarrassment (in this case, as felt by the interviewer).

### Structuring the Dual-Aim Interview

It was mentioned in the introduction that qualitative interviews are pragmatic for exploring potential eHealth working mechanisms as the development or implementation of eHealth programs often entail user interviews anyway. Therefore, researchers who are interested in exploring potential eHealth working mechanisms may do so through existing interviews with applied purposes. However, when applied and basic research goals are mixed like this in the same interview study, it may create an additional challenge in getting rich data on the basic research question. In the study that inspired this paper [24], early interviews had both an applied research goal (getting feedback for improving the program) and a basic research goal (understanding how the participants *related* to the program as a potential eHealth working mechanism). The interview guide started with questions addressing possible sources for program improvement (participants’ likes or dislikes, specific program elements). Toward the end of the interview guide, questions on how the participants related to the program gradually increased in number (*Has the program ever made you happy*? *Have you ever been upset by the program?*). However, most participants answered interview questions on how they related to the program briefly and superficially, resulting in scant data.

Mixing applied and basic research aims can be problematic as different aims may require different interviewing modes. For the interviewer, changing from an *applied* interviewing mode to a *basic* interviewing mode will involve changing the point of his or her focal attention, that is, what to listen for and which follow-up questions to ask. For the participant, changing interviewing modes will involve changing how she or he is expected to answer, from talking more superficially about the breadth of his or her program experiences (*applied mode*) to talking in depth about a few aspects (*basic mode*). If the transition between the different modes is not explicit to the participant, she or he may answer interview questions with the wrong *mindset* —basic interview questions as if they were applied questions or applied interview questions as if they were basic. Unclear transitions may also cause the interviewer to miss important leads in the participant’s answers because of the need to split his or her attention between the 2 research questions. The invisible interaction may exacerbate this challenge: in applied research, the program is treated as a thing, whereas in the search for basic working mechanisms, the program can be considered an interacting agent. An unclear transition between applied and basic research goals may make it more difficult for the participant to take the perspective of the program as an agent influencing the interaction.

Interviews with both applied and basic research aims may serve both aims through topical blocks and clear introductions. The transition can be facilitated by structuring the interview in topical blocks [49]: one covering the applied research question, another covering the basic research question. Topical blocks enable the interviewer to focus on 1 research question at a time, facilitating active listening and choosing following-up questions. The topical blocks should be kept separate; therefore, if the participant says something relevant for research question number 1 in the topical block of research question number 2, the interviewer’s follow-up questions on this should be saved for the respective topical block. Furthermore, the transition between the different topical blocks should be made explicit through small introductions: first, a general introduction to the interview along with a presentation of the topical blocks, then separate introductions preceding each topical block. The introductions can even specify the interviewing modes and what the researcher expects of the participant in each section, for example, that the applied topical block involves factual questions and answers, whereas the basic topical block involves a coresearcher design with joint exploration. Providing the interview with structure and appropriate introductions helps both the participant and the interviewer into the right frame of mind, moving from 1 research question to another. In addition, structuring the dual-aim interview into topical blocks ensures that both research questions are being covered, instead of leaving this overview for analysis.

## Concluding Thoughts

Conducting qualitative interviews is a promising and pragmatic approach for identifying the working mechanisms of automated eHealth programs. Existing user interviews for applied purposes can be used to also pursue basic research questions on eHealth working mechanisms. Researchers planning to conduct user interviews for applied purposes would be wise to ensure the possibility to pursue research questions concerning potential eHealth working mechanisms by including this purpose in the study information provided to ethics boards and prospective participants. However, getting rich data on eHealth working mechanisms through qualitative interviews may be challenging. In this paper, we suggest that challenges may arise partly due to what we have described as the *invisible interaction*: that eHealth programs affect the program users’ change processes through their content *and* how they influence the person-program interaction, but that their influence on the interaction is largely invisible to the user. We have described 5 interview challenges and suggested tools from qualitative methodology to counteract each challenge. These tools may serve as a step toward a set of guidelines for conducting interview studies on eHealth working mechanisms, with the goal of generating rich data that will improve the quality and reach of the findings. Findings from high-quality interview studies can in turn be used to build more general, theoretical knowledge about the working mechanisms of automated eHealth programs. Through theorizing the general working mechanisms of eHealth interventions, we believe that the next generation of eHealth programs can be developed to fully take advantage of this medium’s potential.

## Abbreviations

eHealth: electronic health
